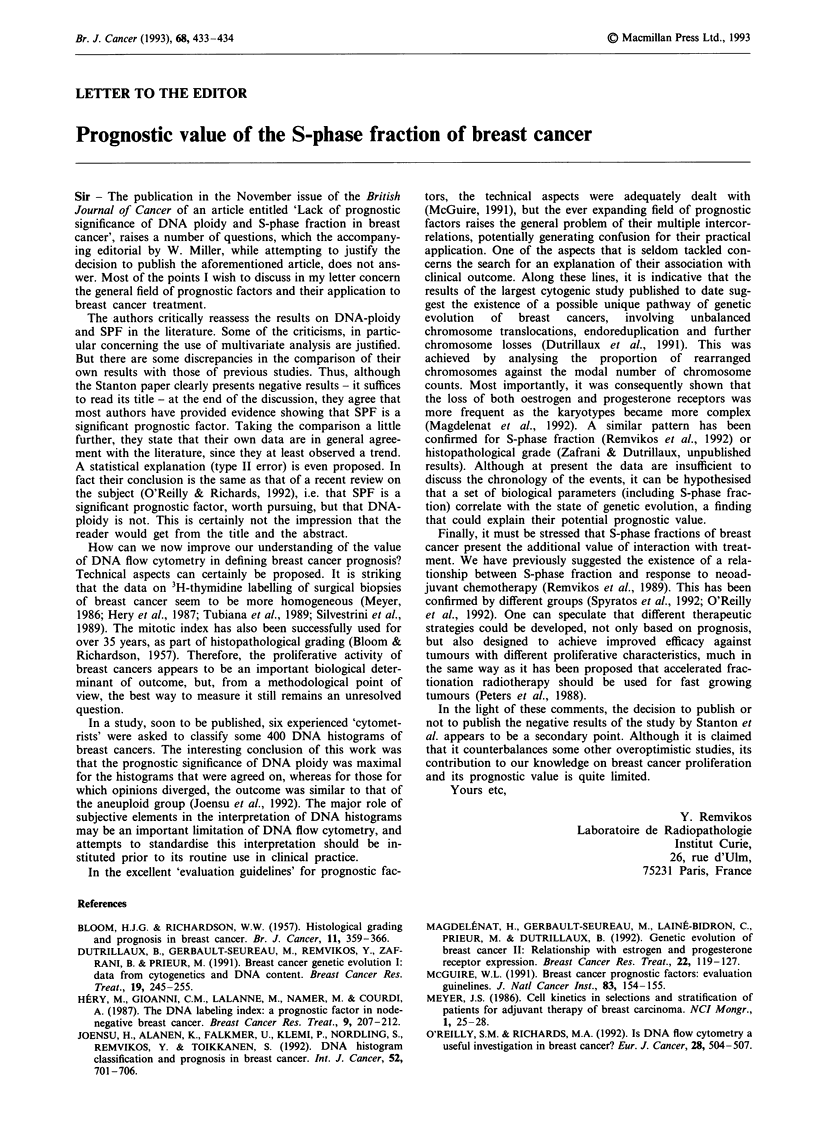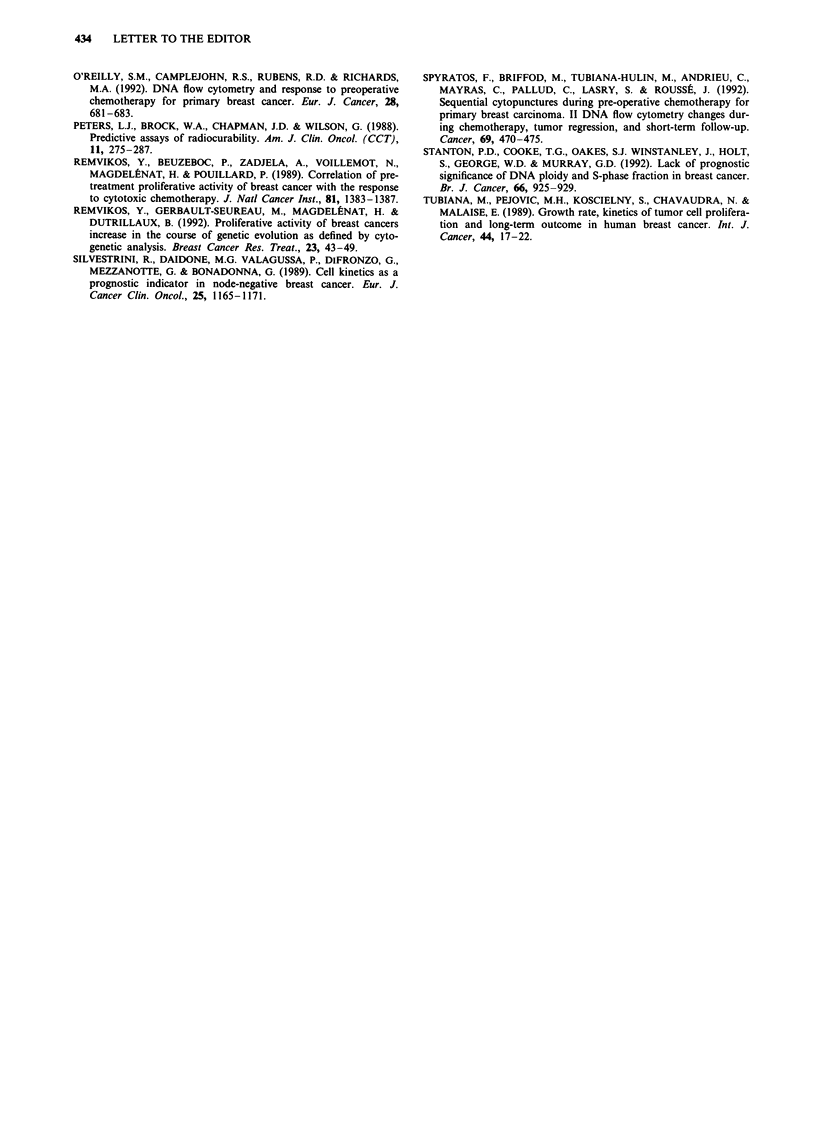# Prognostic value of the S-phase fraction of breast cancer.

**DOI:** 10.1038/bjc.1993.355

**Published:** 1993-08

**Authors:** Y. Remvikos


Br. J. Cancer (1993), 68, 433–434 © Macmillan Press Ltd., 1993

LETTER TO THE EDITOR

### Prognostic value of the S-phase fraction of breast cancer

**Sir** – The publication in the November issue of the *British Journal of Cancer* of an article entitled 'Lack of prognostic significance of DNA ploidy and S-phase fraction in breast cancer', raises a number of questions, which the accompanying editorial by W. Miller, while attempting to justify the decision to publish the aforementioned article, does not answer. Most of the points I wish to discuss in my letter concern the general field of prognostic factors and their application to breast cancer treatment.

The authors critically reassess the results on DNA-ploidy and SPF in the literature. Some of the criticisms, in particular concerning the use of multivariate analysis are justified. But there are some discrepancies in the comparison of their own results with those of previous studies. Thus, although the Stanton paper clearly presents negative results – it suffices to read its title – at the end of the discussion, they agree that most authors have provided evidence showing that SPF is a significant prognostic factor. Taking the comparison a little further, they state that their own data are in general agreement with the literature, since they at least observed a trend. A statistical explanation (type II error) is even proposed. In fact their conclusion is the same as that of a recent review on the subject (O'Reilly & Richards, 1992), i.e. that SPF is a significant prognostic factor, worth pursuing, but that DNA-ploidy is not. This is certainly not the impression that the reader would get from the title and the abstract.

How can we now improve our understanding of the value of DNA flow cytometry in defining breast cancer prognosis? Technical aspects can certainly be proposed. It is striking that the data on $^{3}$H-thymidine labelling of surgical biopsies of breast cancer seem to be more homogeneous (Meyer, 1986; Hery *et al.*, 1987; Tubiana *et al.*, 1989; Silvestrini *et al.*, 1989). The mitotic index has also been successfully used for over 35 years, as part of histopathological grading (Bloom & Richardson, 1957). Therefore, the proliferative activity of breast cancers appears to be an important biological determinant of outcome, but, from a methodological point of view, the best way to measure it still remains an unresolved question.

In a study, soon to be published, six experienced 'cytometrists' were asked to classify some 400 DNA histograms of breast cancers. The interesting conclusion of this work was that the prognostic significance of DNA ploidy was maximal for the histograms that were agreed on, whereas for those for which opinions diverged, the outcome was similar to that of the aneuploid group (Joensu *et al.*, 1992). The major role of subjective elements in the interpretation of DNA histograms may be an important limitation of DNA flow cytometry, and attempts to standardise this interpretation should be instituted prior to its routine use in clinical practice.

In the excellent 'evaluation guidelines' for prognostic factors, the technical aspects were adequately dealt with (McGuire, 1991), but the ever expanding field of prognostic factors raises the general problem of their multiple intercorrelations, potentially generating confusion for their practical application. One of the aspects that is seldom tackled concerns the search for an explanation of their association with clinical outcome. Along these lines, it is indicative that the results of the largest cytogenic study published to date suggest the existence of a possible unique pathway of genetic evolution of breast cancers, involving unbalanced chromosome translocations, endoreduplication and further chromosome losses (Dutrillaux *et al.*, 1991). This was achieved by analysing the proportion of rearranged chromosomes against the modal number of chromosome counts. Most importantly, it was consequently shown that the loss of both oestrogen and progesterone receptors was more frequent as the karyotypes became more complex (Magdelenat *et al.*, 1992). A similar pattern has been confirmed for S-phase fraction (Remvikos *et al.*, 1992) or histopathological grade (Zafrani & Dutrillaux, unpublished results). Although at present the data are insufficient to discuss the chronology of the events, it can be hypothesised that a set of biological parameters (including S-phase fraction) correlate with the state of genetic evolution, a finding that could explain their potential prognostic value.

Finally, it must be stressed that S-phase fractions of breast cancer present the additional value of interaction with treatment. We have previously suggested the existence of a relationship between S-phase fraction and response to neoadjuvant chemotherapy (Remvikos *et al.*, 1989). This has been confirmed by different groups (Spyratos *et al.*, 1992; O'Reilly *et al.*, 1992). One can speculate that different therapeutic strategies could be developed, not only based on prognosis, but also designed to achieve improved efficacy against tumours with different proliferative characteristics, much in the same way as it has been proposed that accelerated fractionation radiotherapy should be used for fast growing tumours (Peters *et al.*, 1988).

In the light of these comments, the decision to publish or not to publish the negative results of the study by Stanton *et al.* appears to be a secondary point. Although it is claimed that it counterbalances some other overoptimistic studies, its contribution to our knowledge on breast cancer proliferation and its prognostic value is quite limited.

Yours etc,

Y. Remvikos
Laboratoire de Radiopathologie
Institut Curie,
26, rue d'Ulm,
75231 Paris, France

**References**

BLOOM, H.J.G. & RICHARDSON, W.W. (1957). Histological grading and prognosis in breast cancer. *Br. J. Cancer*, **11**, 359–366.

DUTRILLAUX, B., GERBAULT-SEUREAU, M., REMVIKOS, Y., ZAFRANI, B. & PRIEUR, M. (1991). Breast cancer genetic evolution I: data from cytogenetics and DNA content. *Breast Cancer Res. Treat.*, **19**, 245–255.

HÉRY, M., GIOANNI, C.M., LALANNE, M., NAMER, M. & COURDI, A. (1987). The DNA labeling index: a prognostic factor in node-negative breast cancer. *Breast Cancer Res. Treat.*, **9**, 207–212.

JOENSU, H., ALANEN, K., FALKMER, U., KLEMI, P., NORDLING, S., REMVIKOS, Y. & TOIKKANEN, S. (1992). DNA histogram classification and prognosis in breast cancer. *Int. J. Cancer*, **52**, 701–706.

MAGDELÉNAT, H., GERBAULT-SEUREAU, M., LAINÉ-BIDRON, C., PRIEUR, M. & DUTRILLAUX, B. (1992). Genetic evolution of breast cancer II: Relationship with estrogen and progesterone receptor expression. *Breast Cancer Res. Treat.*, **22**, 119–127.

MCGUIRE, W.L. (1991). Breast cancer prognostic factors: evaluation guinelines. *J. Natl Cancer Inst.*, **83**, 154–155.

MEYER, J.S. (1986). Cell kinetics in selections and stratification of patients for adjuvant therapy of breast carcinoma. *NCI Mongr.*, **1**, 25–28.

O'REILLY, S.M. & RICHARDS, M.A. (1992). Is DNA flow cytometry a useful investigation in breast cancer? *Eur. J. Cancer*, **28**, 504–507.

O'REILLY, S.M., CAMPLEJOHN, R.S., RUBENS, R.D. & RICHARDS, M.A. (1992). DNA flow cytometry and response to preoperative chemotherapy for primary breast cancer. *Eur. J. Cancer*, **28**, 681–683.

PETERS, L.J., BROCK, W.A., CHAPMAN, J.D. & WILSON, G. (1988). Predictive assays of radiocurability. *Am. J. Clin. Oncol. (CCT)*, **11**, 275–287.

REMVIKOS, Y., BEUZEBOC, P., ZADJELA, A., VOILLEMOT, N., MAGDELÉNAT, H. & POUILLARD, P. (1989). Correlation of pretreatment proliferative activity of breast cancer with the response to cytotoxic chemotherapy. *J. Natl Cancer Inst.*, **81**, 1383–1387.

REMVIKOS, Y., GERBAULT-SEUREAU, M., MAGDELÉNAT, H. & DUTRILLAUX, B. (1992). Proliferative activity of breast cancers increase in the course of genetic evolution as defined by cytogenetic analysis. *Breast Cancer Res. Treat.*, **23**, 43–49.

SILVESTRINI, R., DAIDONE, M.G. VALAGUSSA, P., DIFRONZO, G., MEZZANOTTE, G. & BONADONNA, G. (1989). Cell kinetics as a prognostic indicator in node-negative breast cancer. *Eur. J. Cancer Clin. Oncol.*, **25**, 1165–1171.

SPYRATOS, F., BRIFFOD, M., TUBIANA-HULIN, M., ANDRIEU, C., MAYRAS, C., PALLUD, C., LASRY, S. & ROUSSÉ, J. (1992). Sequential cytopunctures during pre-operative chemotherapy for primary breast carcinoma. II DNA flow cytometry changes during chemotherapy, tumor regression, and short-term follow-up. *Cancer*, **69**, 470–475.

STANTON, P.D., COOKE, T.G., OAKES, S.J. WINSTANLEY, J., HOLT, S., GEORGE, W.D. & MURRAY, G.D. (1992). Lack of prognostic significance of DNA ploidy and S-phase fraction in breast cancer. *Br. J. Cancer*, **66**, 925–929.

TUBIANA, M., PEJOVIC, M.H., KOSCIELNY, S., CHAVAUDRA, N. & MALAISE, E. (1989). Growth rate, kinetics of tumor cell proliferation and long-term outcome in human breast cancer. *Int. J. Cancer*, **44**, 17–22.